# A descriptive analysis of the Fire service Organizational Culture of Safety (FOCUS) survey: FOCUS 3.0 survey wave

**DOI:** 10.1186/s40621-026-00682-5

**Published:** 2026-05-28

**Authors:** Madison E. Raposa, Ashley M. Geczik, Alexandra B. Fisher, Victoria H. Gallogly, Jin Lee, Brisa N. Sánchez, Sandra L. Bloom, Suzy B. Gulliver, Jennifer A. Taylor

**Affiliations:** 1 Department of Environmental and Occupational Health, Drexel University, Dornsife School of Public Health, Philadelphia, USA; 2 Department of Psychological Sciences, Kansas State University, Manhattan, USA; 3 Department of Biostatistics, Drexel University, Dornsife School of Public Health, Philadelphia, United States; 4 Department of Health Management and Policy, Drexel University, Dornsife School of Public Health, Philadelphia, USA; 5 Baylor Scott & White Health, Warriors Research Institute, Waco, USA

**Keywords:** Firefighter, Mental health, Safety climate

## Abstract

**Background:**

The Fire service Organizational Culture of Safety (FOCUS) survey, is an industry-specific assessment tool used to examine aspects of safety climate and leadership, safety behaviors, organizational outcomes, and mental health outcomes. The FOCUS survey provides participating U.S. fire departments with critical information to improve their work environment. The current iteration of the survey (FOCUS 3.0) includes expanded leadership and mental health modules.

**Methods:**

This study consisted of a convenience sample of 9,078 firefighters nested within 89 fire departments (career, combination, volunteer) who assessed with the FOCUS 3.0 survey wave. A descriptive epidemiologic analysis of the FOCUS 3.0 survey metrics was conducted. We examined individual-level and department-level characteristics for the participating departments as well as average department scores. Pearson correlation matrices identified potential relationships between the survey metrics.

**Results:**

Of the 9,078 individuals included in our analysis, the majority were male (88.3%), straight (89.7%), non-officers (66.1%), and non-veterans (83.4%). Our sample consisted of majority career departments (67.4%), with an average of 11 stations, and more than 5,000 calls per year (67.4%). Supervisor support for safety scores were on average 14-points higher than management commitment to safety scores. On average, 12% of respondents endorsed depression symptoms, 10% of respondents reported having anxiety symptoms, and 9% of respondents reported having suicidal ideations within the past month. We observed high positive correlations between management commitment to safety and four leadership metrics (participation in decision making, leadership communication, safety-specific transformational leadership, team psychological safety). Additionally, management commitment to safety was highly negatively correlated with emotional exhaustion (burnout), intent to leave the profession, and mental health metrics.

**Conclusions:**

The findings from this study of FOCUS 3.0 departments are consistent with prior FOCUS waves (beta-test, 1.0, and 2.0). This study identified that the newly added leadership metrics are highly correlated with safety climate metrics. Thus, they may be avenues for departments to improve their organizational safety climate. This study provides an important first look into the proportion of firefighters who participated in FOCUS 3.0 that experience mental health symptoms. Future research should further investigate the mental health metrics and relationships with safety climate.

## Background

Working in the fire service involves repeated exposure to high-stakes and potentially traumatic situations that require coordination among many individuals to address the problem safely and effectively [1]. In addition to these situations, firefighters face other occupational stressors, such as long shifts with limited time for rest and recovery, which can impact an individual’s mental and physical health [2–4]. Therefore, it is crucial to cultivate a work environment that promotes trust, camaraderie, and open communication where individuals can rely on one another. This can help an organization to foster a positive safety climate, organizational safety climate refers to individuals’ perceptions of the organization’s safety policies, procedures, and practices [5]. Safety climate has been identified as one of the strongest predictors of safety behaviors, organizational outcomes, and injuries. Safety climate can be viewed as a resource that can mitigate the impact of a highly demanding occupation based on the Job Demands-Resources (JD-R) theory, which states that high levels of job demands in combination with low job resources results in negative outcomes, such as burnout [6, 7]. Thus, fostering a positive safety climate can serve as a protective factor against burnout and adverse psychological outcomes [6–8].

The Fire service Organizational Culture of Safety (FOCUS) survey is a safety climate assessment tool tailored specifically for U.S. fire departments [9, 10]. The survey includes four domains: safety climate and leadership, safety behavior and compliance, organizational outcomes, and mental health and well-being outcomes. It was developed based on the theories of Christian et al. (2009) and Huang et al. (2016), which found a positive association between safety climate and safety behaviors, and a negative association with safety outcomes, such as injuries [11, 12]. Previous research using data from the FOCUS beta-test survey supported these theories, revealing positive associations between safety climate metrics and safety behaviors, organizational outcomes, and safety outcomes [10, 13]. Similar findings were observed in studies using the FOCUS 1.0 and 2.0 data [14].

The primary aim of this study was to conduct a descriptive analysis of the FOCUS 3.0 survey metrics, with a particular emphasis on the leadership and mental health modules, using a subset of the FOCUS 3.0 survey data. Additionally, we explored the relationships between various survey metrics to understand how they are correlated. We hypothesized that the safety climate predictors, management commitment to safety and supervisor support for safety will be highly positively correlated with outcomes such as job satisfaction and engagement, while negatively correlated with emotional exhaustion (hereafter referred to as burnout) and the mental health metrics.

## Methods

### Population

The analytic sample used for this study is a subset of the first wave of the FOCUS 3.0 survey [April 2023-January 2024, *n* = 89 departments, 9,078 individuals], The second wave is currently in the field.

Data is collected at the individual-level through voluntary online surveys and aggregated to the department level. The FOCUS 3.0 survey wave consisted of 10,022 individuals nested within 90 fire departments. The following exclusionary criteria were used to derive the analytic sample: respondents who were not between the ages of 18 and 85 years or had a missing age (*n* = 394) and respondents who did not respond to all mental health scale items (*n* = 537). One entire department was excluded as all demographic information for their respondents was left blank. The final analytic sample consisted of 9,078 individuals nested within 89 fire departments.

### FOCUS survey overview and metrics

**Fig. 1:**
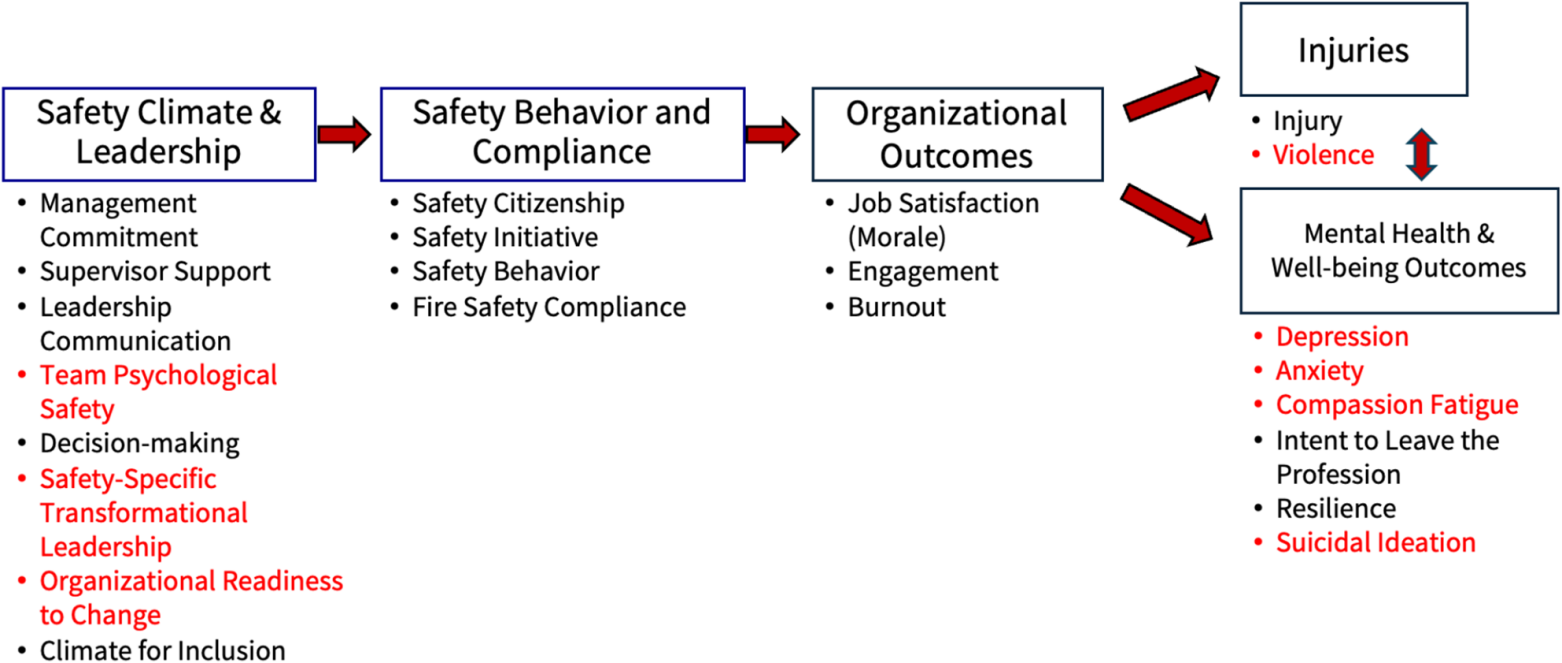
FOCUS Conceptual Pathway. Adapted from Demerouti E et al. [6], Christian et al. [11], Huang et al. [12], and Taylor et al. [10]

The FOCUS survey is a fire service industry-specific assessment tool designed to measure safety climate and its associated outcomes [9, 10]. The conceptual framework guiding our study recognizes organizational safety climate as the primary upstream predictor of organizational outcomes, injuries, and mental health and well-being outcomes (Fig. 1).

To date, there have been four versions of the FOCUS survey, with the most recent being FOCUS 3.0. Each iteration has been updated with additional scales to measure constructs requested by the fire service. Prior waves of the FOCUS survey, its design, and psychometric properties have been previously described [9, 10, 13, 14]. FOCUS 3.0 includes two new modules: (1) the leadership module, which covers team psychological safety, safety-specific transformational leadership, organizational readiness for change, and climate for inclusion; and (2) the mental health module, which includes scales for depression, anxiety, and suicidal ideation. Metrics related to safety climate and leadership, such as management commitment to safety and supervisor support for safety, have been identified as key upstream predictors of organizational outcomes, including injuries. The metrics highlighted in red are new additions to the FOCUS survey. A list of all the scales included on the FOCUS 3.0 survey included in this analysis can be found in Table 1 and are described in the following sections by domain.

**Table 1:** Metrics on the FOCUS 3.0 Survey examined in this study

**Safety Climate & Leadership**	**Management Commitment to Safety** (Taylor et al., 2019)
	**Supervisor Support for Safety** (Taylor et al., 2019)
	**Leadership Communication** (Arnold et al., 2000) **Safety Specific Transformational Leadership** (Kelloway et al., 2006)
	**Organizational Readiness for Implementing Change (ORIC)** (Shea et al., 2014)
	**Climate for Inclusion** (Nishii, 2013)
	**Team Psychological Safety** (Edmondson, 1999)
	**Participation in Decision Making** (Siegel & Ruh, 1973)
**Safety Behavior & Compliance**	**Fire Safety Compliance** (Taylor et al., 2019)
	**Safety Citizenship** (novel - FIRST Center)
	**Safety Initiative** (Kark et al., 2015)
	**Safety Behavior** (novel - FIRST Center)
**Organizational Outcomes**	**Job Satisfaction** (Sexton et al., 2006)
	**Engagement** (Schaufeli, 2002)
	**Emotional Exhaustion (burnout)** (Maslach & Jackson, 1981)
**Injuries**	**Injury**
**Mental Health & Well-being Outcomes**	**Depression (PHQ-9)** (Kroenke et al., 2001)
	**Anxiety (GAD-7)** (Spitzer et al., 2006)
	**Compassion Fatigue** (Adams et al., 2008)
	**Intent to Leave the Profession** (Blauuw et al., 2013)
	**Resilience** (Campbell-Sills & Stein, 2007)
	**Suicidal Ideation Attributes Scale (SIDAS)** (Van Spijker et al. 2014)

### Safety climate & leadership

The FOCUS survey measures safety climate with two validated scales: management commitment to safety and supervisor support for safety. Management commitment to safety is defined as member’s perceptions of how departmental leadership values and supports safety within their department [10]. Supervisor support for safety refers to member’s perceptions of their direct supervisor’s commitment to safety within their crew or group [10]. Management commitment to safety and supervisor support for safety are both measured using 7-items. Response options for these two metrics were on a 5-point Likert scale (strongly disagree to strongly agree).

We measured six additional aspects of safety climate and leadership: leadership communication, organizational readiness for implementing change, climate for inclusion, safety-specific transformational leadership scale, participation in decision making, and team psychological safety. Leadership communication, measured using 6-items, is defined as the degree to which leadership disseminates important information to their members [15]. Organizational readiness for implementing change (ORIC), measured using 12-items, captures how employees of an organization feel about their ability to implement change in their department [16]. Climate for inclusion, measured using 15-items, refers to the inclusivity of an individual’s work environment defined through fair implemented employment practices, integration of differences, and inclusion in decision making [17]. The response options for these three metrics were on a 5-point Likert scale (strongly disagree to strongly agree). The safety-specific transformational leadership scale, measured using 10-items, assesses how department leaders influence safety through four dimensions: idealized influence, inspirational motivation, intellectual stimulation, and individualized consideration. Response options range from “not at all” to “frequently or always” on a 5-point Likert scale [18]. Participation in decision making, measured using 7-items, is defined as the degree to which leadership includes members in decision making processes in the department. Response options range from “never” to “always” on a 5-point Likert scale [19]. Team psychological safety, measured using 7-items, refers to members’ beliefs about interpersonal risk-taking. Response options range from “very inaccurate” to “very accurate” on a 7-point scale [20].

### Safety behavior & compliance

The safety behavior and compliance domain consist of four scales: safety citizenship, safety behavior, safety initiative, and fire safety compliance. Safety citizenship, measured using 3-items, assesses firefighters’ proactive and voluntary actions aimed at improving safety, with response options ranging from “never” to “always” on a 5-point Likert scale (novel - FIRST Center). Safety behavior, measured using 4-items, assesses firefighters’ safety awareness and practices, such as wearing seatbelts when traveling to and from calls, with response options ranging from “strongly disagree” to “strongly agree” on a 5-point scale (novel – FIRST Center). Safety initiative, measured using 7-items, is defined as individual employee’s change-oriented behaviors towards a safer working environment [21]. We measure this initiative on a 5-point Likert scale, with response options ranging from “to a very slight extent” to “to a very large extent.” Fire safety compliance, measured using 4-items, measures the degree to which members act accordingly with safety protocols and processes on a fire run, with response options ranging from “never” to “always” on a 5-point Likert scale [10].

### Organizational outcomes

Organizational outcomes, including burnout, engagement, and job satisfaction, were included on the FOCUS survey, and asked twice in relation to work on fire runs versus EMS (emergency medical service) runs. Burnout, defined by Maslach’s Burnout Inventory, refers to feelings of being emotionally overextended or exhausted by one’s work [22]. The 9-items response options ranged from “never” to “always” on a 5-point Likert scale. For burnout, a lower score is desired. Engagement, measured using 6-items, refers to the work-related state characterized by vigor, absorption, and dedication [23]. Response options ranged from “never” to “always” on a 5-point Likert scale. Job satisfaction, measured using 4-items, refers to the degree of positivity about work [24]. Response options ranged from “strongly disagree” to “strongly agree”.

### Mental health & well-being outcomes

The mental health and well-being domain measures six scales posited to be outcomes of safety climate: compassion fatigue, resilience, intent to leave the profession, depression, anxiety, and suicidal ideation. Compassion fatigue, composed of two components, job burnout and secondary trauma, refers to hazards faced by first responders following traumatic events [25]. We used 13-items (8 items for job burnout and 5 items for secondary trauma) with response options ranged from “never/rarely” and “very often” on a 5-point Likert scale. Resilience, measured using 10-items, assesses an individual’s ability to positively adapt when faced with stress and cope with adversity [26]. Response options ranged from “not at all true” to “true nearly all the time” on a 5-point Likert scale. Intent to leave the profession, or one’s desire to leave the fire service in the near future, is measured using one survey item and is separately in regard to fire and EMS work [27]. Individuals were asked to independently think about whether they desire to leave their current fire role and their current EMS role. Lower scores for compassion fatigue and intent to leave the profession are desired as a higher score indicates a worse outcome.

The three mental health metrics from FOCUS 3.0 are screening tools used to determine whether individuals may be experiencing symptoms of these mental health conditions. Depression, measured using the Patient Health Questionnaire-9 (PHQ-9), assesses the degree of depression symptom severity [28]. We categorized depression using a dichotomous measure indicating a positive screen for depression symptoms (composite score ≥ 10). Response options ranged from “not at all” to “nearly every day” on a 5-point Likert scale. Anxiety was measured using the Generalized Anxiety Disorder-7 (GAD-7) and screens individuals for generalized anxiety disorder and assesses symptom severity [29]. Like depression, we used a categorization method indicating a positive screen for anxiety symptoms (composite score ≥ 10) was used. Response options ranged from “not at all” to “nearly every day” on a 4-point scale (scored 0–3). We used the Suicidal Ideation Attributes Scale (SIDAS) to assess five attributes of suicidal ideation: frequency, controllability, closeness to attempts, distress, and interference with daily living activities [30]. The scale begins with the question “In the past month, how often have you had thoughts about suicide?” with responses ranging from 0 (Never) to 10 (Always), with unlabeled points between. If participants select “Never” (0), they skip the remaining four items. A composite score of the sum of all items is calculated. We categorized ideation severity into no suicidal ideation (0) versus any suicidal ideation (1–50). These mental health metrics are not intended to diagnose individuals with psychological conditions as that falls under the expertise of clinicians.

### Metric scoring

To improve interpretability by the fire and rescue service, scores for all metrics (except mental health) were converted to a 100-point scale. For example, an individual respondent’s management commitment score of 4.7 on the 5-point scale is equivalent to a 94 on the 100-point scale. Individual responses were aggregated and reported as a department-level score.

To provide a benchmark for individual departments, possible scores were broken into three categories. For variables in which a higher score is desired, a score of greater than or equal to 80 was defined as being within the “maintenance zone”. The cut-point of 80 was used as it equates to scores of “agree” (4) or “strongly agree” (5) on the Likert scale or a positive response. Scores between 61 and 79 were defined as “opportunities for improvement.” Finally, scores below 60 were defined as “areas of concern.” The cut-points for the other two categories were not decided on by empirical evidence, but to provide practical benchmarks for departments. These categories provide departments with a way to prioritize areas that may need change or maintenance. However, for burnout, intention to leave the profession, and compassion fatigue, a lower score is desired and a cut-point of less than or equal to 40 was defined as being within the “maintenance zone.”

### Descriptive variables

Individual demographic characteristics including age, years of experience, sex assigned at birth, gender identity, sexual orientation, race and ethnicity, education, rank, and active-duty veteran status were collected.

Department characteristics were collected during the enrollment process. These variables included the number of stations, roster size, annual number of calls, population served, and Federal Emergency Management Agency (FEMA) region.

### Statistical analysis

We reported individual-level (age, years of experience, sex assigned at birth, gender identity, sexual orientation, race and ethnicity, officer status, education, veteran status, and injury) and department-level (number of stations, roster size, annual number of calls, population served, FEMA region, organization type) descriptive statistics. For continuous variables the mean, standard deviation (SD) and range were reported. For categorical variables, counts and percentages were reported.

We calculated mean scores for each metric at both the department level and across the entire sample. For depression, anxiety, and suicidal ideation, individual sum scores for each metric were used to indicate whether an individual “screened” positive for the condition. Department level values were indicated by the percent of individuals who “screened” positive for each condition divided by the total roster size of the department. The minimum, maximum, and mean percentages were reported. Pearson correlation matrices were produced to identify correlations between metrics on the individual level. All statistical analyses were conducted using R Statistical Software (v 4.3.1) and R Studio [31, 32]. This research received Institutional Review Board (IRB) approval by the University IRB.

## Results

Our analytic sample consisted of 9,078 individuals nested within 89 fire departments of four different organization types: career (67.4%), combination-mostly career (23.6%), combination-mostly volunteer (6.7%), and volunteer (2.2%). Departments who assessed with the FOCUS 3.0 survey had on average 11 stations (SD 14.9), more than 5,000 calls per year (67.4%), and served more than 50,000 people (62.9%). The response rate for departments ranged from 6% to 104%, with an average of 57%. Of our sample, 49%) of departments (*n* = 44) had a department-level response rate of 60% or higher (results not shown). Additional department-level characteristics are reported in Table 2.

**Table 2:** Fire Department Characteristics of FOCUS 3.0 Analytic Sample

	Total departments (*n* = 89)	
	Mean ± SD	Min-Max
**Number of Stations**	11 ± 14.9	1–71
	Mean	Min-Max
**Response Rate**	57%	6%-104%
	*N*	*%*
**Organization Type**		
Volunteer	2	2.2
Combination - Mostly Volunteer	6	6.7
Combination - Mostly Career	21	23.6
Career	60	67.4
**Roster Size**		
0–49	23	25.8
50–149	34	38.2
150+	32	36.0
**Annual Number of Calls**		
0-4999	29	32.6
5000–9999	18	20.2
10,000–49,999	25	28.1
50,000+	17	19.1
**Population Served**		
5000–9999	8	9.0
10,000–24,999	10	11.2
25,000–49,999	15	16.9
50,000–99,999	20	22.5
100,000+	36	40.4
**FEMA Region**		
1	5	5.6
2	3	3.4
3	8	9.0
4	15	16.9
5	14	15.7
6	10	11.2
7	6	6.7
8	10	11.2
9	6	6.7
10	12	13.5
**CPSE accreditation**		
Yes	23	25.8
No	66	74.2
**ISO Rating**		
High (1, 2, 3)	71	79.8
Medium (4, 5, 6)	13	14.6
Low (7, 8, 9, 10)	5	5.6

Of the 9,078 individuals included in our analysis, respondents on average were 39.8 years old (SD 10.0) had 16.1 years of experience (Table 3). The majority of respondents were male (88.3%), straight (89.7%), non-officers (66.1%), and non-veterans (83.4%). In the past 12 months, 1,923 individuals reported being injured (21.2%) and 1,233 of those received medical treatment (64.1%) as a result.

**Table 3:** Individual-level Descriptive Characteristics of FOCUS 3.0 Analytic Sample

	Total Respondents (*N* = 9078)	
	Mean ± SD	Min-Max
**Age**	39.8 ± 10.0	18–85
**Years of Experience**	16.1 ± 39.6	0–58
	*N*	*%*
**Sex assigned at birth**		
Female	516	5.7
Male	8,015	88.3
Prefer not to say	486	5.4
Missing	61	0.7
**Sexual Orientation**		
Lesbian or gay	89	1.0
Straight	8,142	89.7
Bisexual	80	0.9
Something else	82	0.9
Prefer not to say	599	6.6
Missing	86	0.9
**Gender Identity**		
Man	8,008	88.2
Woman	478	5.3
Trans Male/Trans Man	9	0.1
Trans Female/Trans Woman	10	0.1
Non-Binary/Genderqueer/Gender Non-Conforming	48	0.5
Different Identity	26	0.3
Prefer not to say	455	5.0
Missing	44	0.5
**Officer Status** ^**1**^		
Non-officer (Firefighter, EMT, Paramedic)	5,997	66.1
Officer (Lieutenant, Captain)	2,431	26.8
Leadership (Battalion Chief, Chief, Commissioner)	650	7.2
**Ethnicity**		
Non-Hispanic	8,441	93
Hispanic	637	7.0
**Race** ^**2**^		
American Indian or Alaskan Native	71	0.8
Asian	59	0.7
Black or African American	612	6.7
Native Hawaiian or Pacific Islander	28	0.3
White	6,566	72.3
Other	214	2.4
Multiracial	225	2.4
Prefer not to say	805	8.9
Missing	498	5.5
**Education**		
Less than high school	13	0.1
High school or equivalent	3,205	35.3
College degree	5,009	55.2
Graduate degree	498	5.5
Missing	353	3.9
**Veteran Status**		
Not a Veteran	7,573	83.4
Veteran	1,200	13.2
Missing	305	3.4
**Injury last 12 months**		
No	7,109	78.3
Yes	1,923	21.2
Missing	46	0.5
**Medical Treatment for Injury**		
No	626	32.6
Yes	1,233	64.1
Missing	64	3.3

^1^ For rank, a three-level categorical variable was created with the following categories: non-officer (firefighter, paramedic, EMT), company officer (lieutenant, captain), leadership (battalion chief, chief, commissioner). Individuals that selected more than one rank were classified by the highest rank reported

^2^ Race/ethnicity were asked with the option to “select all that apply.” If an individual selected more than one response for race/ethnicity, they were categorized as “multiracial.”

Management commitment to safety scores for the sample had a 50-point range (42–92) with a mean of 69 (Table 4). Supervisor support for safety scores had a much narrower range (75–92) with a mean of 83. Leadership communication, safety-specific transformational leadership, organizational readiness for implementing change, climate for inclusion, and team psychological safety all had scores averaging between 64 and 76. While all these metrics had similar scores hovering around 70, a few had large ranges in their scores. Of note, participation in decision making had the lowest score of 59 and a large range of 28 to 83.

**Table 4:** Department-level FOCUS Survey Results

Domain	Scale	Min	Avg. Dept. Score	Max
**Safety Climate and Leadership**	Management Commitment to Safety	42	**69**	92
	Supervisor Support for Safety	75	**83**	92
	Leadership Communication	39	**67**	88
	Safety-Specific Transformational Leadership	31	**67**	90
	Organizational Readiness for Implementing Change	61	**76**	91
	Climate for Inclusion	38	**70**	91
	Team Psychological Safety	52	**64**	79
	Participation in Decision Making	28	**59**	83
**Safety Behavior and Compliance**	Fire Safety Compliance	80	**89**	100
	Safety Citizenship	78	**86**	100
	Safety Initiative	57	**66**	82
	Safety Behavior	74	**87**	96
**Organizational Outcomes**	Emotional Exhaustion (Burnout) - EMS	33	**43**	62
	Emotional Exhaustion (Burnout) - Fire	24	**38**	47
	Engagement - EMS	62	**73**	84
	Engagement - Fire	72	**83**	90
	Job Satisfaction - EMS	58	**74**	91
	Job Satisfaction - Fire	70	**84**	95
**Mental Health and Well-Being Outcomes**	Resilience	76	**82**	88
	Intention to Leave to the Profession - EMS	23	**37**	62
	Intention to Leave to the Profession - Fire	23	**36**	56
	Compassion Fatigue - Job Burnout	27	**36**	47
	Compassion Fatigue - Secondary Trauma	27	**36**	47
		Min	**Avg. Dept. %**	Max
	Depression	0%	**12%**	43%
	Anxiety	0%	**10%**	43%
	Suicidal Ideation	0%	**9%**	33%

For the safety behavior and compliance module, fire safety compliance, safety citizenship, and safety behaviors all had mean scores around 87, with moderately small ranges. However, safety initiative had a mean score of 66 with scores ranging from 57 to 82.

Within the organizational outcomes domain, we observed a 5-point higher average score for burnout on EMS was observed compared to fire runs (43 vs. 38). The maximum burnout on EMS score (62) was 15-points higher than the maximum score on fire runs (47). For engagement and job satisfaction, where higher scores are desired, a 10-point higher average score for engagement and job satisfaction on fire compared to EMS runs was noted.

For the mental health and well-being outcomes module, we observed a resilience score of 82 with a tight range of 76 to 88. Intent to leave the profession was nearly the same on EMS and fire runs with average scores around 36 and a moderate range. Compassion fatigue job burnout and secondary trauma had identical mean scores of 36 (range: 27–47).

Our sample had an average of 12% of department membership endorsing depression symptoms with values ranging from 0% to 43%. Of this sample, an average of 10% of department membership reported having anxiety symptoms (0% to 43%). Additionally, an average of 9% of department membership reported having suicidal ideation within the past month, with some departments having as high as 33% of members endorsing symptoms.

**Fig. 2:**
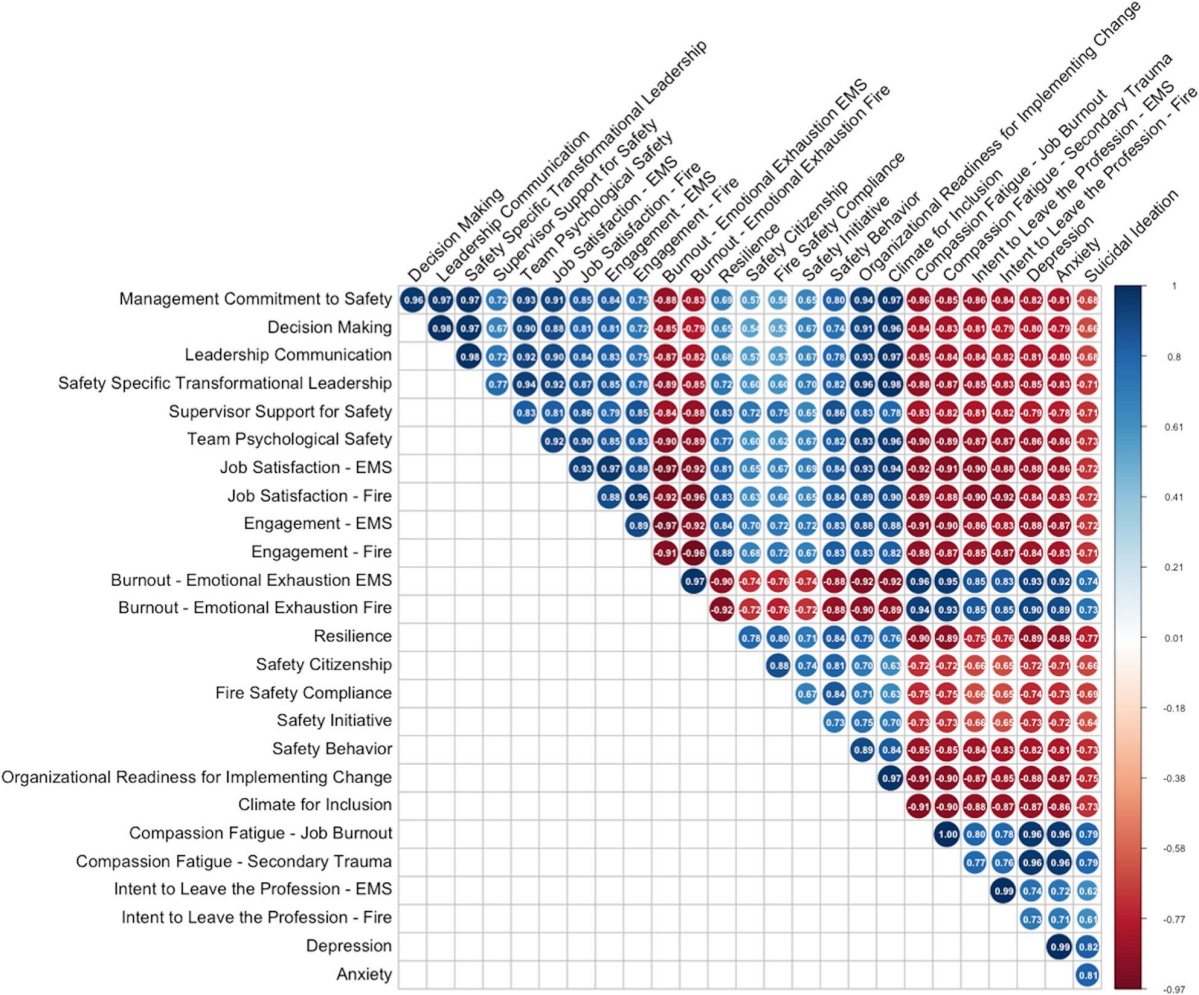
Individual-level Pearson Correlation Coefficient Matrices for FOCUS 3.0 Metrics

The Pearson correlation matrices revealed that management commitment to safety is highly positively correlated (correlation coefficient (*r)* > = 0.80) with ten metrics (Fig. 2): participation in decision making (*r* = 0.96), leadership communication (*r* = 0.97), safety-specific transformational leadership (*r* = 0.97), team psychological safety (*r* = 0.93), job satisfaction on EMS (*r* = 0.91), job satisfaction on fire (*r* = 0.85), engagement on EMS (*r* = 0.84), safety behavior (*r* = 0.80), ORIC (*r* = 0.94), and climate for inclusion (*r* = 0.97). Management commitment to safety is moderately positively correlated (*r* between 0.50 and 0.79) with six metrics: supervisor support for safety (*r* = 0.72), engagement on fire (*r* = 0.75), resilience (*r* = 0.69), safety citizenship (*r* = 0.57), fire safety compliance (*r* = 0.58), and safety initiative (*r* = 0.65). Additionally, management commitment to safety is highly negatively correlated with burnout on EMS (*r* = -0.88), burnout on fire (*r* = -0.83), intent to leave the profession on both EMS and Fire (*r* = -0.86 and *r* = -0.84 respectively), and two of the mental health metrics [depression (*r* = -0.82), anxiety (*r* = -0.81)]. Further, management commitment to safety is moderately negatively correlated with suicidal ideation (*r* = -0.68).

The Pearson correlation matrices revealed that supervisor support for safety is highly positively correlated (correlation coefficient (*r)* > = 0.80) with seven metrics (Fig. 2): team psychological safety (*r* = 0.83), job satisfaction on EMS (*r* = 0.81), job satisfaction on fire (*r* = 0.86), engagement on fire (*r* = 0.85), resilience (*r* = 0.83), safety behavior (*r* = 0.86), and ORIC (*r* = 0.83). Management commitment to safety is moderately positively correlated (*r* between 0.50 and 0.79) with nine metrics: management commitment to safety (*r* = 0.72), participation in decision making (*r* = 0.67), leadership communication (*r* = 0.72), safety-specific transformational leadership (*r* = 0.77), engagement on EMS (*r* = 0.79), safety citizenship (*r* = 0.72), fire safety compliance (*r* = 0.75), and safety initiative (*r* = 0.65), and climate for inclusion (*r* = 0.78). Additionally, supervisor support for safety is highly negatively correlated with burnout on EMS (*r* = -0.84), burnout on fire (*r* = -0.88), intent to leave the profession on both EMS and Fire (*r* = -0.81 and *r* = -0.82 respectively). Further, supervisor support for safety is moderately negatively correlated with depression (*r* = -0.79), anxiety (*r* = -0.78), and suicidal ideation (*r* = -0.71).

## Discussion

The objective of this study was to conduct a descriptive analysis of the FOCUS 3.0 survey, with particular emphasis on the newly incorporated leadership and mental health modules. Our study results were consistent with prior studies using FOCUS survey data [9, 10, 13, 14]. Most notably, management commitment to safety scores were on average lower than supervisor support for safety scores, with management commitment scores having a wider range. This is thought to be the result of decreased interaction between individuals and department leadership. The results of our correlation analyses found that there are several metrics that are positively correlated with management commitment to safety. Thus, these may be areas on which departments can improve.

This study highlights metrics that are highly variable between different departments. Within the safety climate and leadership domain, leadership communication, safety-specific transformational leadership, climate for inclusion, and participation in decision-making have wide ranges and overall low or “needs improving” scores. In the safety behavior and compliance domain, all metrics are similar except for safety initiative. This indicates that individuals are following the instructions and policies provided by their department, but likely do not voluntarily take steps (e.g., trying new approaches to improving workplace safety; improving safety policies and practices) to improve safety while at work. Future studies should investigate the reasoning behind the lower scores observed for this metric.

The inclusion of mental health scales in the FOCUS 3.0 survey was a novel addition, implemented in response to concerns expressed by fire service partners regarding the mental well-being of their members. This study allowed for the prevalence estimates for depression, anxiety, and suicidal ideation in a large sample of firefighters from fire departments across the United States. When comparing with other general population estimates, our study revealed a higher percentage of individuals with depression (9% versus 12%, respectively) and suicidal ideation (5% versus 9%, respectively) [33, 34]. However, our estimate of anxiety is similar to that within the general population (10% versus 12.5%, respectively) [34]. Generally, the fire and rescue service are a highly resilient population and resilience is known to improve an individual’s ability to deal with depression and anxiety. Yet, in our highly resilient sample, the prevalence of depression and suicidal ideation outweigh the general population. It is important that this relationship is investigated in future studies to determine ways to reduce the prevalence of these mental health conditions.

Prior research of mental health in the fire service has resulted in varying proportions of mental health symptoms. This is likely due to several factors including the use of varying scales to measure mental health symptoms [1, 34–38]. One study by Stanley et al. found that 46.8% of individuals endorsed having suicidal ideation, which is much larger than general population estimates and what was observed in our study [37]. However, it is important to note that a different suicidal ideation scale was utilized. A modified version of the Self-Injurious Thoughts and Behaviors Interview – Short Form (SITBI-SF) was used to assess career prevalence of suicidal ideation among career and retired firefighters. This scale assesses suicidal ideation by asking “Since becoming a firefighter, have you ever had thoughts of killing yourself?” The data collected via this question can be threatened by recall bias as it is asking individuals to remember potentially decades of time. Our study used the suicidal ideation attributes scale (SIDAS), which asks about suicidal ideation in the past month. This poses a much easier time-period for individuals to remember, reducing the potential concern of recall bias. Another example is in a study of depression and PTSD by Gulliver et al., where the Beck Depression Inventory for Primary Care (BDI-PC) was used to measure depression among participants [39]. They found very few individuals exhibited depression symptoms and were subsequently diagnosed with depression. The sample size for this study was considerably smaller than the one presented in this study. To compare results across different studies, it would be useful for researchers to harmonize the scales that are used to measure mental health in the fire and rescue service, as well as the recall periods. To gain a true understanding of what mental health trends look like in the fire service, longitudinal studies using the same scales over time should be completed. This will also allow for researchers to understand if there are fluctuations in mental health symptoms within this occupation and identify factors that may be related such as call volume, traumatic events, or departmental changes.

Framing the overall results within the JD-R theory, departments within our sample had relatively low management commitment to safety scores, when management commitment to safety is generally viewed as a resource for individuals can draw from to promote positive outcomes. However, the lack of departmental support accompanied by the high demands of working in the fire and rescue service, it is unsurprising that we observed the levels of burnout, job satisfaction, intent to leave the profession, and mental health outcomes that we did. Thus, to improve mental health and job satisfaction it is imperative that departments address areas, such as safety climate, that can mitigate the impacts of the highly demanding job of a firefighter and EMS provider.

### Strengths and limitations

One notable strength of this study is the large and diverse sample size of 89 fire departments inclusive of 9,078 firefighters. In contrast to prior investigations that have been limited to small numbers of departments, restricted geographic regions, or small participant numbers, this study encompasses fire departments from all ten FEMA regions, representing both urban and rural environments with varying roster sizes, and all three organization types (career, combination, and volunteer) [1, 34–38].

While the study has several strengths, it also has important limitations to acknowledge. First, the sample is a convenience sample, which may introduce potential selection bias, as departments voluntarily enroll to participate in the FOCUS assessment. These departments may be more safety-oriented, have prior experience with such assessments, or be especially motivated to improve their safety culture. However, the data exhibits significant heterogeneity, with wide ranges in scores across different metrics. This indicates that the study is capturing a diverse array of departments, both “optimal” and “suboptimal,” rather than a uniform set of high-performing organizations. This lowers our concern the impact of selection bias. The demographic characteristics of our sample are consistent with previous waves of FOCUS survey waves and representative of the broader U.S. fire and rescue service with the mean age of respondents being 40, 5% of the sample being female, and the majority of individuals having more than 10 years of experience [40].

## Conclusions

This study expands on prior findings of the FOCUS survey data through analysis of new leadership metrics on FOCUS 3.0 that are highly correlated with safety climate metrics. Thus, departments are encouraged to focus on or make changes to their organization in an effort to improve their organizational safety climate. This study also emphasizes the importance of mental health screening to understand the state of mental health in the fire and rescue service. Future research should further investigate the mental health metrics and relationships with safety climate.

## Data Availability

Due to IRB limitations, the datasets analyzed during this study are not publicly available but are available from the corresponding author upon reasonable request.

## Abbreviations

FOCUS: Fire Service Organizational Culture of Safety survey
FEMA: Federal Emergency Management Agency
JD-R: Job Demands-Resources
ORIC: Organizational Readiness for Implementing Change
EMS: Emergency medical services
PHQ-9: Patient Health Questionnaire 9
GAD-7: Generalized Anxiety Disorder 7
SIDAS: Suicidal Ideation Attributes Scale
SD: Standard deviation
EMT: Emergency medical technician
CPSE: Center for Public Safety Excellence
ISO: Insurance Services Office
SITBI-SF: Self-Injurious Thoughts & Behaviors Interview – Short Form
BDI-PC: Beck Depression Inventory for Primary Care

## References

[1] Johnson CC, Vega L, Kohalmi AL, Roth J, Howell BR, Hasselt VBV. Enhancing mental health treatment for the firefighter population: Understanding fire culture, treatment barriers, practice implications, and research directions. *Professional Psychology: Research and Practice*. Published online 2020. https://api.semanticscholar.org/CorpusID:210583961

[2] Patterson PD, Weaver MD, Frank RC, et al. Association Between Poor Sleep, Fatigue, and Safety Outcomes in Emergency Medical Services Providers. Prehospital Emerg Care. 2012;16(1):86–97. 10.3109/10903127.2011.616261. 10.3109/10903127.2011.616261 PMC3228875 22023164

[3] Paterson JL, Aisbett B, Ferguson SA. Sound the alarm: Health and safety risks associated with alarm response for salaried and retained metropolitan firefighters. Saf Sci. 2016;82:174–81. 10.1016/j.ssci.2015.09.024.

[4] Billings JM, Haddock CK, Jahnke SA. Intra-Tour Variation of Firefighter Sleep Duration and Sleep-Wake Cycle within the 24/48 and 48/96 Shift Schedules. Behav sleep Med. 2023;21(1):1–12. 10.1080/15402002.2021.2021912. 34989296 10.1080/15402002.2021.2021912

[5] Zohar D. Safety climate in industrial organizations: Theoretical and applied implications. J Appl Psychol. 1980;65(1):96–102. 10.1037/0021-9010.65.1.96. 7364709

[6] Demerouti E, Bakker AB, Nachreiner F, Schaufeli WB. The job demands-resources model of burnout. J Appl Psychol. 2001;86(3):499–512. 10.1037/0021-9010.86.3.499. 11419809

[7] Demerouti E, Bakker AB. The Job Demands–Resources model: Challenges for future research. SA J Industrial Psychol. 2011;37(2):2. 10.4102/sajip.v37i2.974.

[8] Lee J, Resick CJ, Allen JA, Davis AL, Taylor JA. Interplay between Safety Climate and Emotional Exhaustion: Effects on First Responders’ Safety Behavior and Wellbeing Over Time. J Bus Psychol. 2024;39(1):209–31. 10.1007/s10869-022-09869-1. 10.1007/s10869-022-09869-1 PMC9772603 36573129

[9] Davis AL, Allen J, Shepler L, et al. Moving FOCUS – The Fire Service Organizational Culture of Safety survey – From research to practice. J Saf Res. 2020;74:233–47. 10.1016/j.jsr.2020.06.011. 10.1016/j.jsr.2020.06.011 32951788

[10] Taylor JA, Davis AL, Shepler LJ, et al. Development and validation of the fire service safety climate scale. Saf Sci. 2019;118:126–44. 10.1016/j.ssci.2019.05.007.

[11] Christian MS, Bradley JC, Wallace JC, Burke MJ. Workplace safety: A meta-analysis of the roles of person and situation factors. J Appl Psychol. 2009;94(5):1103–27. 10.1037/a0016172. 19702360 10.1037/a0016172

[12] Huang YH, Lee J, McFadden AC, et al. Beyond safety outcomes: An investigation of the impact of safety climate on job satisfaction, employee engagement and turnover using social exchange theory as the theoretical framework. Appl Ergon. 2016;55:248–57. 10.1016/j.apergo.2015.10.007. 26611987 10.1016/j.apergo.2015.10.007

[13] Geczik AM, Lee J, Davis AL, Allen JA, Taylor JA. Size matters: How safety climate and downstream outcomes vary by fire department organization type. Injury Epidemiol. 2022;9(1):11. 10.1186/s40621-022-00373-x. 10.1186/s40621-022-00373-x PMC8941800 35321756

[14] Geczik AM, Lee J, Allen JA, et al. An updated analysis of safety climate and downstream outcomes in two convenience samples of U.S. fire departments (FOCUS 1.0 and 2.0 survey waves). Inj Epidemiol. 2024;11(1):19. 10.1186/s40621-024-00502-8. 38773566 10.1186/s40621-024-00502-8 PMC11106928

[15] Arnold JA, Arad S, Rhoades JA, Drasgow F. The empowering leadership questionnaire: the construction and validation of a new scale for measuring leader behaviors. J Organiz Behav. 2000;21(3):249–69. 10.1002/(SICI)1099-1379(200005)21:3<249::AID-JOB10>3.0.CO2.

[16] Shea CM, Jacobs SR, Esserman DA, Bruce K, Weiner BJ. Organizational readiness for implementing change: a psychometric assessment of a new measure. Implement Sci. 2014;9(1):7. 10.1186/1748-5908-9-7. 24410955 10.1186/1748-5908-9-7 PMC3904699

[17] Nishii LH. The Benefits of Climate for Inclusion for Gender-Diverse Groups. AMJ. 2013;56(6):1754–74. 10.5465/amj.2009.0823.

[18] Kelloway EK, Mullen J, Francis L. Divergent effects of transformational and passive leadership on employee safety. J Occup Health Psychol. 2006;11(1):76–86. 10.1037/1076-8998.11.1.76. 16551176 10.1037/1076-8998.11.1.76

[19] Siegel AL, Ruh RA. Job involvement, participation in decision making, personal background and job behavior. Organizational Behav Hum Perform. 1973;9(2):318–27. 10.1016/0030-5073(73)90055-X.

[20] Edmondson A. Psychological Safety and Learning Behavior in Work Teams. Adm Sci Q. 1999;44(2):350–83. 10.2307/2666999.

[21] Kark R, Katz-Navon T, Delegach M. The dual effects of leading for safety: The mediating role of employee regulatory focus. J Appl Psychol. 2015;100(5):1332–48. 10.1037/a0038818. 25664472 10.1037/a0038818

[22] Maslach C, Jackson SE. The measurement of experienced burnout. J Organ Behav. 1981;2(2):99–113. 10.1002/job.4030020205.

[23] Schaufeli WB, Salanova M, González-romá V, Bakker AB. The Measurement of Engagement and Burnout: A Two Sample Confirmatory Factor Analytic Approach. J Happiness Stud. 2002;3(1):71–92. 10.1023/A:1015630930326.

[24] Sexton JB, Helmreich RL, Neilands TB, et al. The Safety Attitudes Questionnaire: psychometric properties, benchmarking data, and emerging research. BMC Health Serv Res. 2006;6(1):44. 10.1186/1472-6963-6-44. 16584553 10.1186/1472-6963-6-44 PMC1481614

[25] Adams RE, Figley CR, Boscarino JA. The Compassion Fatigue Scale: Its Use With Social Workers Following Urban Disaster. Res Social Work Pract. 2008;18(3):238–50. 10.1177/1049731507310190. 10.1177/1049731507310190 PMC2367230 18458750

[26] Campbell-Sills L, Stein MB. Psychometric analysis and refinement of the connor–davidson resilience scale (CD‐RISC): Validation of a 10‐item measure of resilience. J Trauma Stress. 2007;20(6):1019–28. 10.1002/jts.20271. 18157881 10.1002/jts.20271

[27] Blaauw D, Ditlopo P, Maseko F, et al. Comparing the job satisfaction and intention to leave of different categories of health workers in Tanzania, Malawi, and South Africa. Global Health Action. 2013;6(1):19287. 10.3402/gha.v6i0.19287. 23364090 10.3402/gha.v6i0.19287 PMC3556679

[28] Kroenke K, Spitzer RL, Williams JBW. The PHQ-9: Validity of a brief depression severity measure. J Gen Intern Med. 2001;16(9):606–13. 10.1046/j.1525-1497.2001.016009606.x. 11556941 10.1046/j.1525-1497.2001.016009606.x PMC1495268

[29] Spitzer RL, Kroenke K, Williams JBW, Löwe B. A Brief Measure for Assessing Generalized Anxiety Disorder: The GAD-7. Arch Intern Med. 2006;166(10):1092. 10.1001/archinte.166.10.1092. 16717171 10.1001/archinte.166.10.1092

[30] Van Spijker BAJ, Batterham PJ, Calear AL, et al. The Suicidal Ideation Attributes Scale (SIDAS): Community-Based Validation Study of a New Scale for the Measurement of Suicidal Ideation. Suicide Life Threat Behav. 2014;44(4):408–19. 10.1111/sltb.12084. 24612048 10.1111/sltb.12084

[31] Posit Team. RStudio: Integrated Development Environment for R. Published online 2023. http://www.posit.co/

[32] R Core Team. R: A language and environment for statistical computing. Published online 2023. https://www.R-project.org

[33] Substance Abuse and Mental Health Services Administration. (2023). Key substance use and mental health indicatorsin the United States: Results from the 2022 National Survey on Drug Use and Health (HHS Publication No.PEP23-07-01-006, NSDUH Series H-58). Center for Behavioral Health Statistics and Quality, Substance Abuse andMental Health Services Administration. https://www.samhsa.gov/data/report/2022-nsduh-annual-national-report

[34] Norris T, Adjaye-Gbewonyo D, Bottoms-McClain L. Early Release of Selected Estimates Based on Data From the 2023 National Health Interview Survey. National Center for Health Statistics; 2023.

[35] Garmon-Jones L, Hanna P, John M. A systematic review of the factors that contribute towards mental health in the fire service. Int J Emerg Serv. 2023;12(2):125–44. 10.1108/IJES-02-2021-0005.

[36] Jahnke SA, Poston WSC, Haddock CK, Murphy B. Firefighting and mental health: Experiences of repeated exposure to trauma. Work. 2016;53(4):737–44. 10.3233/WOR-162255. 26890595 10.3233/WOR-162255

[37] Stanley IH, Hom MA, Hagan CR, Joiner TE. Career prevalence and correlates of suicidal thoughts and behaviors among firefighters. J Affect Disord. 2015;187:163–71. 10.1016/j.jad.2015.08.007. 26339926 10.1016/j.jad.2015.08.007

[38] Testoff AC, Pauley JL, Brewer M et al. Mental Health Disorders, Organizational Stigma, and Health Service Utilization among U.S. Fire Investigators: A Cross-Sectional Survey. *Journal of occupational and environmental medicine*. Published online 2024. https://api.semanticscholar.org/CorpusID:270923140 10.1097/JOM.0000000000003173 38955804

[39] Gulliver SB, Zimering RT, Knight JA, et al. A prospective study of firefighters’ PTSD and depression symptoms: The first 3 years of service. Psychol trauma: theory Res Pract policy. 2021;13 1:44–55. https://api.semanticscholar.org/CorpusID:229929537. 10.1037/tra0000980 33382330

[40] Fahy R, Evarts B, Stein G. *US Fire Department Profile 2020*. National Fire Protection Association (NFPA); 2022. https://content.nfpa.org/-/media/Project/Storefront/Catalog/Files/Research/NFPA-Research/Emergency-responders/osfdprofile.pdf?rev=11492dd98ca94be580c0dab13e4500ef

